# Incremental prognostic value of myocardial strain in patients with coronary slow flow

**DOI:** 10.1186/s12880-026-02241-2

**Published:** 2026-02-21

**Authors:** Binyu Zhou, Peixuan Shi, Wenhui Song, Weizong Wang, Haiyan Wang

**Affiliations:** 1 Department of Medical Ultrasound, The First Affiliated Hospital of Shandong First Medical University & Shandong Provincial Qianfoshan Hospital, Jinan, China; 2 Department of Health Management Center, Cheeloo College of Medicine, Qilu Hospital, Shandong University, Jinan, China; 3 Department of Medical Ultrasound, Weifang Yidu Central Hospital, Weifang, China; 4 Department of Cardiology, The First Affiliated Hospital of Shandong First Medical University & Shandong Provincial Qianfoshan Hospital, Jinan, China

**Keywords:** Coronary slow flow, Prognostic value, Major adverse cardiovascular events, Speckle tracking imaging, Echocardiography, Myocardial strain

## Abstract

**Background:**

Although coronary slow flow (CSF) was associated with adverse cardiac outcomes, the prognostic value of myocardial strain (MS) for major adverse cardiovascular events (MACEs) in CSF is not well established. We aimed to examine the longitudinal association between MS and MACEs in this population.

**Methods:**

Medical records from 336 patients with CSF (January 2019 to June 2022) were analyzed retrospectively. All patients underwent comprehensive echocardiography with MS quantified via speckle tracking echocardiography (STE), including left ventricular global longitudinal strain (LVGLS) and circumferential strain (LVGCS). MACEs were defined as the primary endpoint. Multivariable Cox regression analysis was performed to evaluate the association between MS and MACEs.

**Results:**

During a median follow-up of 19 months (range: 6–51 months), MACEs occurred in 37.8% (127/336). LVGCS was independently associated with MACEs (HR = 1.794, 95% CI 1.307–2.462, *P* < 0.001) after adjusting for conventional risk factors and may provide incremental prognostic value beyond conventional risk factors (C statistics increased from 0.790 to 0.885; IDI = 0.172, *P* < 0.001; NRI = 0.790, *P* < 0.001).

**Conclusions:**

LVGCS was independently associated with MACEs in CSF patients. Adding LVGCS to conventional risk factors may provide incremental prognostic information, suggesting its potential utility for improving risk stratification.

## Introduction

Coronary slow flow (CSF), a prevalent coronary microvascular disorder, is defined as delayed distal perfusion on angiography without significant epicardial stenosis (> 50%) [1]. Occurring in 5–24% of chest pain patients undergoing coronary angiography (CAG) for suspected CHD [2]. Angina pectoris is the most common clinical presentation of CSF patients, which affects their quality of life and even leads to ventricular tachyarrhythmias or cardiac death [3]. For patients with CSF, identifying those at high risk of adverse events is critical to guide targeted interventions and improve prognosis. However, reliable markers to identify high-risk CSF patients remain lacking in clinical practice, highlighting the urgency of exploring effective risk stratification tools for targeted interventions.

Conventional cardiovascular risk factors (including age, sex, smoking, hypertension, and diabetes) and established risk stratification tools (including the Framingham Risk Score and Pooled Cohort Equations) are cornerstones of cardiovascular risk prediction [4, 5]. However, these approaches exhibit notable limitations in the context of CSF. Myocardial strain (MS) derived from speckle-tracking echocardiography (STE) has emerged as a sensitive indicator of subclinical myocardial impairment. Beyond its utility in detecting coronary heart disease (CHD) and assessing post-revascularization myocardial remodeling, MS confers robust prognostic value in CHD patients [6]. While MS is well-established for predicting CSF and evaluating myocardial function [7], its potential prognostic significance in patients with CSF has not been systematically investigated to date.

Thus, this study aims to explore the relationship between MS and MACEs, and further assess whether MS could provide incremental prognostic value beyond conventional risk factors in predicting MACEs among patients with CSF.

## Materials and methods

### Study population

This retrospective study analyzed inpatients with CSF diagnosed by CAG at the First Affiliated Hospital of Shandong First Medical University from January 2019 to June 2022.

The diagnostic criteria for CSF are as follows: (1) there is no coronary artery stenosis or stenosis ≤ 40% at CAG, and (2) the coronary flow rate measured by the thrombolysis in myocardial infarction (TIMI) frame count (TFC) (or corrected TFC) higher than 27 frames (30 frames/s) in at least one epicardial coronary artery [8].

Patients with coronary artery spasm, coronary artery dilatation, prior revascularization, congenital heart disease, cardiomyopathy, significant valvular disease, autoimmune diseases, tumors, systemic disease, loss to follow-up, or poor echocardiographic image quality were excluded.

### Coronary arteriography

The standard Judkins technique was used for CAG in all subjects. Angiography was performed at 30 frames/second. TFC calculates the number of exposure frames in which the contrast medium enters the target vessel and develops distally [9]. The TFC values of the left anterior descending coronary artery (LAD) and left circumflex (LCX) were evaluated in the right anterior oblique projections, whereas the TFC values of the right coronary artery (RCA) were acquired in the left anterior oblique projections. Because LAD is usually longer than LCX and RCA, the TFC of LAD should be divided by 1.7 to obtain the corrected TFC (cTFC) [9]. Coronary flow was evaluated by two experienced cardiologists who were blinded to the clinical information of each participant.

To assess the inter-observer agreement for CSF diagnosis, angiographic images of 30 randomly selected subjects were independently re-evaluated by the same two cardiologists who performed the initial assessments. Both reviewers were blinded to each other’s evaluations and to the clinical data. Any disagreements were resolved by consensus, with adjudication by a third senior cardiologist if consensus could not be reached.

### Clinical and laboratory data

Based on the literature, after the discussion at the expert meeting, a total of 7 clinical indicators and 12 laboratory indicators were included as possible risk factors, including conventional cardiovascular risk factors (including age, sex, smoking, hypertension, and diabetes), drinking, hyperlipidemia (HLP), red blood cell count (RBC), hemoglobin (HGB), mean platelet volume (MPV), D-dimer (DD), fibrinogen (Fb), serum alkaline phosphatase (ALP), albumin (Alb), high-density lipoprotein (HDL), low-density lipoprotein (LDL), lipoprotein (Lp), homocysteine (Hcy), blood glucose (Glu).

### Echocardiographic acquisition and analysis

All echocardiographic examinations (Epiq 7 C, Philips Healthcare) were performed within 24 hours after CAG. Conventional echocardiographic measurements were performed according to the guidelines of the American Society of Echocardiography and the European Association of Cardiovascular Imaging [10]. Left atrium anterior-posterior diameter (LAD) and left ventricular end-diastolic diameter (LVEDD) were assessed via 2D measurements. The left ventricular ejection fraction (LVEF) was measured via the modified biplane Simpson method. Doppler was used to measure the peak early-diastolic (E) velocity and peak tricuspid regurgitation (TR) velocity. The peak early diastolic velocities (e’) of the mitral annulus at both the lateral and septal walls were acquired via tissue Doppler imaging, and the average value was used to calculate E/e’. Pulmonary artery systolic pressure (PASP) was estimated to be 4× (peak TR velocity) ^2^ + right atrium pressure.

2D STE images of the left ventricle were obtained from 3 parasternal short-axis views and 3 apical views, and were analyzed using the 2D Cardiac Performance Analysis package of Tom Tec software (version 2.31). Left ventricular global longitudinal strain (LVGLS) and global circumferential strain (LVGCS) were semi-automatically analyzed along the endocardium. This process began with manual delineation of the left ventricular endocardial border, after which the system automatically tracked these borders frame-by-frame throughout the cardiac cycle. Individual tracings or points were manually corrected wherever incorrect. The segments with poor tracking quality were not included in the analysis. All MS values are reported as percentage change from baseline, derived by averaging segmental strain values across all left ventricular segments and imaging views. These procedures were completed by two experienced echocardiographers who were blinded to all clinical data.

Reproducibility of LVGLS and LVGCS was evaluated by inter-observer and intra-observer variability analyses. Thirty randomly selected subjects were reanalyzed by one echocardiographer (intra-observer) after > 4 weeks and independently analyzed by a second echocardiographer (inter-observer), with blinding to previous results.

### Follow-up and endpoints

Follow-up was conducted via telephone calls and outpatient visits using a standardized questionnaire to assess chest tightness or chest pain, palpitation, cerebrovascular events, all-cause mortality, and major adverse cardiac events (MACEs). The primary endpoint of the study was MACEs, which included cardiac death, heart failure, stroke, nonfatal myocardial infarction (MI), severe arrhythmia (e.g., second-degree or higher atrioventricular block, paroxysmal or persistent atrial fibrillation, sick sinus syndrome, frequent ventricular premature beats, and ventricular fibrillation), and rehospitalization for angina. Patients were divided into two groups according to the presence or absence of MACEs.

Cardiac death was defined as death due to cardiac origins. Heart failure was diagnosed according to the ESC heart failure guidelines [11]. Stroke was confirmed by evidence of ischemic cerebral infarction due to thrombotic or embolic occlusion [12]. Nonfatal MI was diagnosed in the presence of characteristic signs and symptoms suggesting myocardial ischemia with serial changes in cardiac biomarkers of myocardial damage [13]. Rehospitalization for angina was defined as readmission due to recurrent angina attacks.

### Sample size determination

Sample size was determined based on the events-per-variable (EPV) rule for Cox regression. With 11 independent variables and an EPV of 10–20, a minimum of 110–220 MACEs was required (10 × 11 to 20 × 11). Based on a predicted MACE incidence of 35% from a previous study [2], the estimated sample size was 314–629 participants (110–220 ÷ 0.35). A total of 336 participants were enrolled, and 127 MACEs occurred during follow-up, yielding an EPV of 11.5, which falls within the recommended range of 10–20. Sensitivity analysis showed that even with a clinically plausible MACE incidence of 30%–40%, the EPV remained above 10, supporting the statistical reliability of the model.

### Statistical analysis

Normally distributed data are expressed as mean ± standard deviation (SD) and were compared using the independent sample t-test. Non-normally distributed data are presented as median (25%, 75%) and were compared via the Mann–Whitney U test. Categorical data are reported as n (%) and were compared via the χ² test or Fisher’s exact test. Given that missing data for baseline indicators were < 10%, multiple imputation was performed to address these values, and the resultant findings were validated using sensitivity analysis with complete case analysis.

Cox proportional hazards models were used to identify independent predictors of MACEs, with results reported as hazard ratios (HRs) and 95% confidence intervals (CIs). Variables with *P* < 0.10 in univariate Cox regression analysis, combined with conventional risk factors (including age, sex, smoking, hypertension, and diabetes), were included in the multivariable stepwise Cox regression models. Stepwise selection was performed with forward entry (*P* < 0.05) and backward removal (*P* > 0.10). The proportional hazards assumption was verified via Schoenfeld residuals (plots and significance tests). Potential multicollinearity was assessed by Spearman’s correlation and variance inflation factor (VIF) for all model variables.

The predictive value of MS for MACEs was evaluated using receiver operating characteristic (ROC) curve analysis, with the area under the curve (AUC) calculated. Discriminative ability was assessed using the C statistic for each Cox regression model. In addition, the net reclassification index (NRI) and integrated discrimination improvement (IDI) were calculated to assess the predictive improvement of MS beyond conventional risk factors.

Reproducibility of MS measurements was quantified using the intraclass correlation coefficient (ICC) and Bland–Altman plots. Inter-observer agreement for the diagnosis of CSF was assessed using Cohen’s kappa coefficient (κ). Two-tailed *P* < 0.05 was considered statistically significant for all parameters. Statistical analyses were performed using the SPSS software for Windows, version 26 (IBM Corp, Armonk, NY), and R version 3.5.2 (R Foundation for Statistical Computing, Vienna, Austria).

## Results

### Study population

**Fig. 1:**
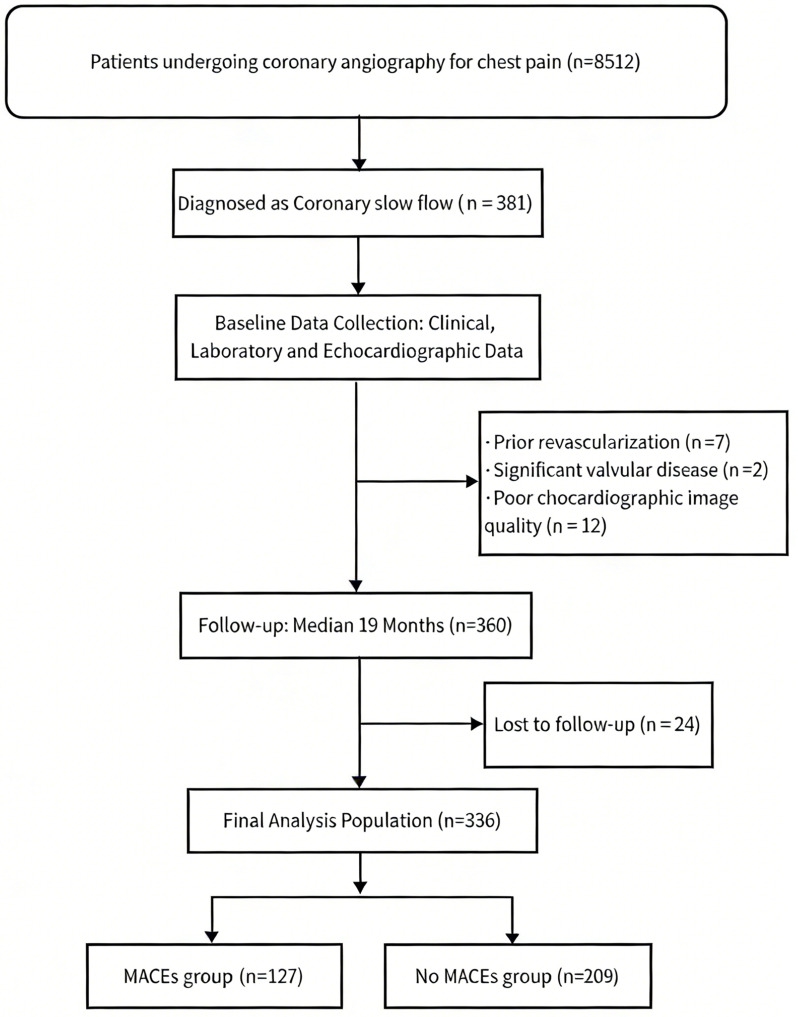
Patient screening and grouping flowchart

A total of 8512 patients who underwent CAG between January 2019 and June 2022 were initially enrolled in this retrospective study. All participants presented with clinical indications for CAG, including chest pain, chest pressure, or dyspnea suggestive of angina. Among them, 381 were diagnosed with CSF. Subsequently, 7 patients with a history of prior revascularization, 2 with significant valvular heart disease, 24 lost to follow-up, and 12 with inadequate echocardiographic image quality were excluded. Ultimately, 336 CSF patients were included in the final analysis (Fig. 1).

### Follow-up

During a median follow-up of 19 months (range 6–51 months), 127 patients (37.8%) in the CSF cohort developed MACEs. The composite endpoint comprised: cardiac death (*n* = 3, 0.9%), heart failure hospitalization (*n* = 14, 4.2%), stroke (*n* = 12, 3.6%), nonfatal myocardial infarction (*n* = 22, 6.5%), severe arrhythmias requiring intervention (*n* = 26, 7.7%), and angina-related rehospitalization (*n* = 50, 14.9%). Concurrently, symptom reports included chest tightness (*n* = 122, 36.3%), chest pain (*n* = 85, 25.3%), palpitations (*n* = 78, 23.2%), chronic arrhythmias (*n* = 18, 5.4%), and cerebrovascular events (*n* = 6, 1.8%).

### Reproducibility of MS measurements

**Fig. 2:**
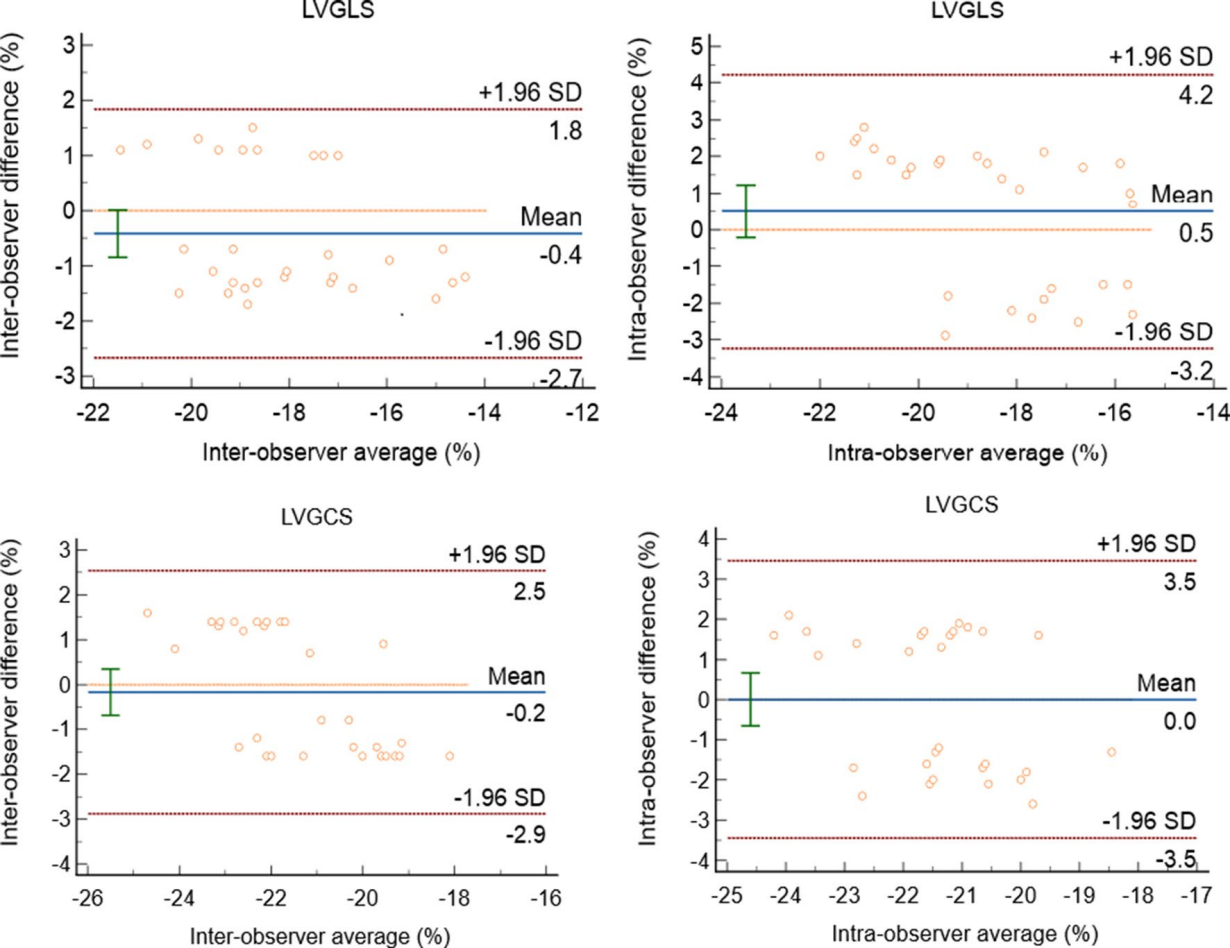
Bland-Altman analyses for the intra- and inter-observer variability of LVGLS and LVGCS. LVGLS, left ventricular global longitudinal strain; LVGCS, left ventricular global circumferential strain

Intra- and inter-observer reproducibility for LVGLS and LVGCS were high (Fig. 2). ICCs confirmed excellent reproducibility of MS: intra-observer ICCs were 0.919 (95% CI: 0.839–0.961) for LVGLS and 0.909 (95% CI: 0.819–0.956) for LVGCS, while inter-observer ICCs were 0.819 (95% CI: 0.654–0.910) and 0.895 (95% CI: 0.791–0.949), respectively.

### Inter-observer agreement for CSF diagnosis

The inter-observer agreement for the diagnosis of CSF was excellent, with a κ value of 0.852 (95% CI: 0.784–0.928), indicating almost perfect agreement. Disagreements occurred in 2 cases (6.7%), both of which were resolved by consensus without requiring adjudication by a third reviewer.

### Differences between the MACEs group and the no MACEs group

Patients with MACEs demonstrated significantly lower LVGCS and LVGLS compared to those without MACEs (all *P* < 0.05). Additionally, these patients were older, had a higher prevalence of HLP, and exhibited lower RBC, HGB, and Alb levels, but higher DD levels, as detailed in Table 1.

**Table 1:** Characteristics of the study cohort

Variables	MACEs (*n* = 127)	No MACEs (*n* = 209)	*P*
Age (year)	62.50 (56.00, 71.00)	55.00 (49.75, 62.00)	< 0.001
Male, n (%)	68 (53.54%)	146 (69.86%)	0.098
Smoking, n (%)	39 (30.71%)	97 (46.41%)	0.097
Drinking, n (%)	41 (32.28%)	92 (44.02%)	0.211
Hypertension, n (%)	49 (38.58%)	97 (46.41%)	0.406
Diabetes, n (%)	10 (7.87%)	17 (8.13%)	0.889
HLP, n (%)	21(16.53%)	29 (13.86%)	0.022
RBC (10^12^/L)	4.50 (4.18, 4.76)	4.72 (4.49, 5.14)	0.014
HGB (g/L)	137.00 (130.25, 147.00)	149.00 (133.50, 154.50)	0.021
MPV (fl.)	10.25 (9.80, 10.90)	10.30 (9.80, 10.90)	0.899
DD (µg/mL)	0.23 (0.17, 0.32)	0.16 (0.11, 0.25)	0.009
Fb (g/L)	2.62 (2.32, 2.96)	2.71 (2.52, 3.15)	0.215
ALP (U/L)	66.00 (55.75, 82.00)	67.00 (55.00, 75.00)	0.961
Alb (g/L)	42.40 (40.43, 45.85)	45.50 (41.80, 48.00)	0.017
HDL (mmol/L)	1.15 (0.93, 1.39)	1.10 (0.97, 1.38)	0.828
LDL (mmol/L)	2.55 (1.88, 3.10)	2.30 (1.92, 2.75)	0.327
Lp (mmol/L)	125.00 (58.00, 267.60)	93.80 (41.15, 118.50)	0.131
Hcy (µmol/L)	11.55 (10.50, 14.65)	12.60 (10.65, 18.55)	0.286
Glu (mmol/L)	5.00 (4.64, 5.51)	5.07 (4.60, 5.50)	0.929
LAD (mm)	36.00 (32.50, 38.00)	34.50 (32.25, 37.00)	0.426
LVEDD (mm)	44.00 (41.00, 47.00)	45.50 (44.00, 47.00)	0.222
LVEF (%)	64.00 (61.00, 68.00)	65.00 (62.50, 70.00)	0.140
PASP (mmHg)	23.50 (20.00, 27.00)	22.00 (19.00, 25.00)	0.265
E (cm/s)	62.00 (50.00, 72.00)	68.00 (58.00, 84.00)	0.065
E/e’	9.50 (8.00, 12.00)	9.35 (8.00, 11.00)	0.530
LVGLS (%)	-18.20 (-18.63, -17.78)	-18.70 (-19.63, -18.08)	0.013
LVGCS (%)	-20.80 (-21.40, -20.38)	-22.25 (-22.90, -21.68)	< 0.001

HLP, hyperlipidemia; RBC, red blood cell count; HGB, hemoglobin; MPV, mean platelet volume; DD, D-dimer; Fb, fibrinogen; ALP, serum alkaline phosphatase; Alb, albumin; HDL, high-density lipoprotein; LDL, low-density lipoprotein; Lp, lipoprotein; Hcy, homocysteine; Glu, blood glucose; LAD, Left atrium anterior-posterior diameter; LVEDD, Left ventricular end-diastolic diameter; LVEF, Left ventricular ejection faction; PASP, pulmonary artery systolic pressure; E, peak early-diastolic velocity; LVGLS, left ventricular global longitudinal strain; LVGCS, left ventricular global circumferential strain

### Univariate and multivariable Cox regression analysis

In univariate analysis, RBC, HGB, ALP, LDL, Hcy, E/e’, LVGLS, and LVGCS were associated with MACEs (all *P* < 0.05). The hazard ratio for LVGCS (HR = 1.727, *P* < 0.001) was higher than that for LVGLS (HR = 1.289, *P* = 0.046). Collinearity analysis revealed a strong correlation between RBC and HGB (*r* = 0.886, *P* < 0.001) and a moderate correlation between LVGLS and LVGCS (*r* = 0.742, *P* < 0.001). Despite these observed correlations, all VIFs were below 3, confirming that multicollinearity was within acceptable limits and did not adversely affect the stability of the regression models.

After adjustment for conventional risk factors by stepwise multivariate Cox regression, ALP and LVGCS were independently associated with MACEs (Table 2). For each 1-unit increase in ALP, the hazard of MACEs increased by 1.9% (HR = 1.019, 95% CI 1.002–1.035). Similarly, each 1% absolute decrease in LVGCS was associated with a 79.4% increase in the hazard of MACEs (HR = 1.794, 95% CI 1.307–2.462). The proportional hazards assumption was satisfied based on Schoenfeld residuals (all *P* > 0.05).

**Table 2:** Factors associated with major adverse cardiac events in all patients

Variables	Univariable			Multivariable		
	HR	95%CI	*P* Valve	HR	95%CI	*P* Valve
Age (year)	1.022	(0.996–1.047)	0.094			
Male	0.661	(0.380–1.149)	1.147			
Smoking	0.779	(0.434–1.344)	0.404			
Drinking	0.769	(0.428–1.384)	0.381			
Hypertension	0.913	(0.525–1.587)	0.746			
Diabetes	0.496	(0.634–3.353)	0.358			
HLP	1.144	(0.447–2.926)	0.076			
RBC (10^12^/L)	0.533	(0.289–0.982	0.044			
HGB (g/L)	0.967	(0.956–0.996)	0.017			
MPV (fl.)	1.128	(0.850–1.499)	0.404			
DD (µg/mL)	1.038	(0.643–2.965)	0.409			
Fb (g/L)	1.336	(0.795–2.243)	0.274			
ALP (U/L)	1.021	(1.005–1.035)	0.009	1.019	(1.002–1.035)	0.024
Alb (g/L)	0.932	(0.865–1.004)	0.063			
HDL (mmol/L)	1.451	(0.625–3.367)	0.386			
LDL (mmol/L)	1.121	(1.001–1.156)	0.048			
Lp (mmol/L)	1.000	(0.999–1.002)	0.562			
Hcy (µmol/L)	0.952	(0.910–0.997)	0.037			
Glu (mmol/L)	0.913	(0.700-1.183)	0.479			
BNP (pg/ml)	1.001	(0.997–1.006)	0.586			
LAD (mm)	1.014	(0.954–1.078)	0.657			
LVEDD (mm)	0.957	(0.883–1.038)	0.291			
IVSd (mm)	1.147	(0.964–1.366)	0.122			
LVEF (%)	1.004	(0.950–1.062)	0.883			
PASP (mmHg)	1.035	(0.964–1.111)	0.337			
E/e’	1.183	(1.058–1.322)	0.003			
LVGLS (%)	1.289	(1.005–1.655)	0.046			
LVGCS (%)	1.727	(1.325–2.251)	< 0.001	1.794	(1.307–2.462)	< 0.001

HLP, hyperlipidemia; RBC, red blood cell count; HGB, hemoglobin; MPV, mean platelet volume; DD, D-dimer; Fb, fibrinogen; ALP, serum alkaline phosphatase; Alb, albumin; HDL, high-density lipoprotein; LDL, low-density lipoprotein; Lp, lipoprotein; Hcy, homocysteine; Glu, blood glucose; LAD, Left atrium anterior-posterior diameter; LVEDD, Left ventricular end-diastolic diameter; LVEF, Left ventricular ejection faction; PASP, pulmonary artery systolic pressure; LVGLS, left ventricular global longitudinal strain; LVGCS, left ventricular global circumferential strain

### Incremental predictive value of MS compared with conventional risk factors for MACEs

**Fig. 3:**
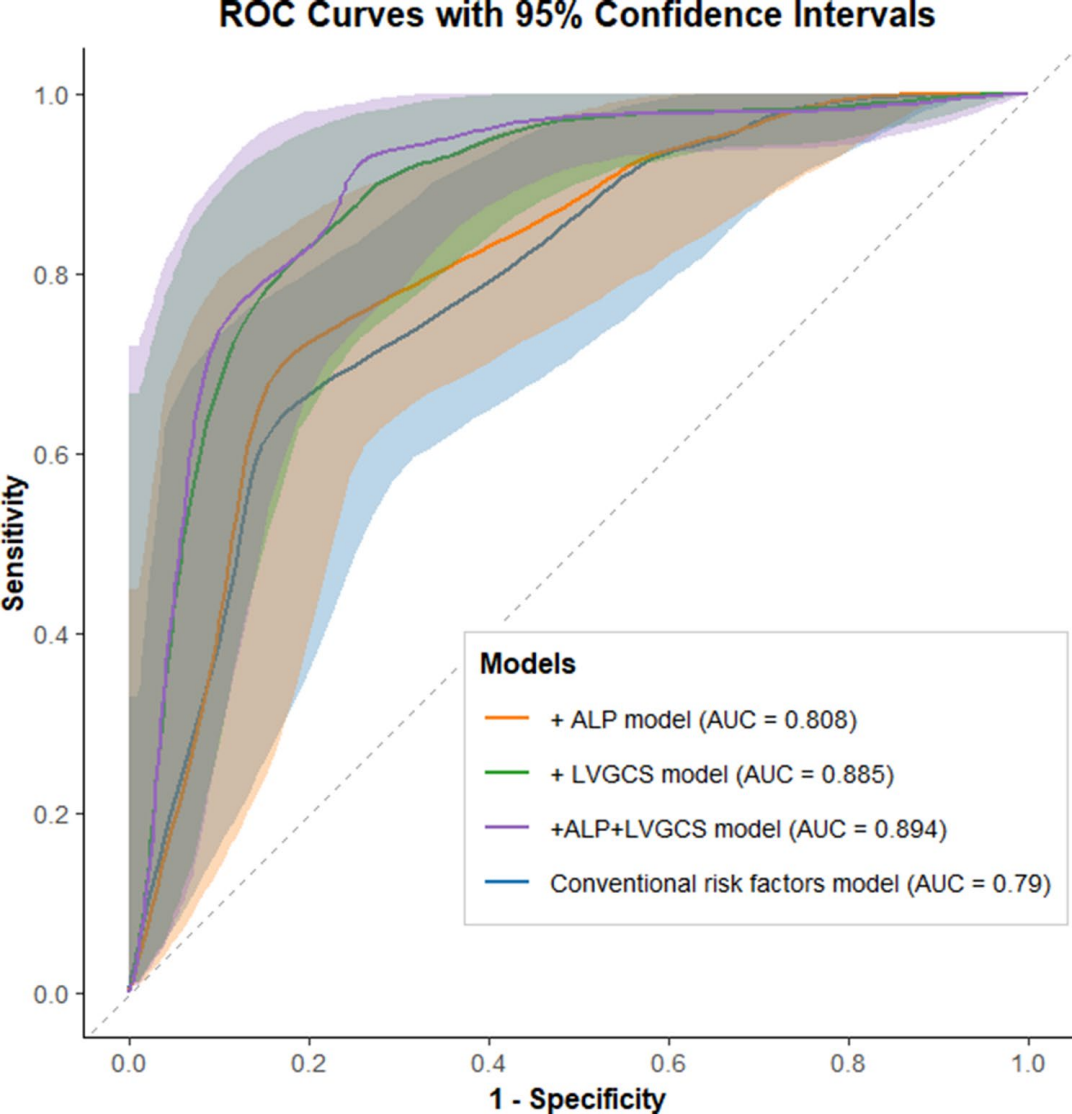
ROC curve analysis of the risk prediction model for MACEs in patients with CSF. AUC, area under the curve; ALP, serum alkaline phosphatase; LVGCS, left ventricular global circumferential strain; Conventional risk factors, including age, sex, smoking, hypertension, and diabetes

**Fig. 4:**
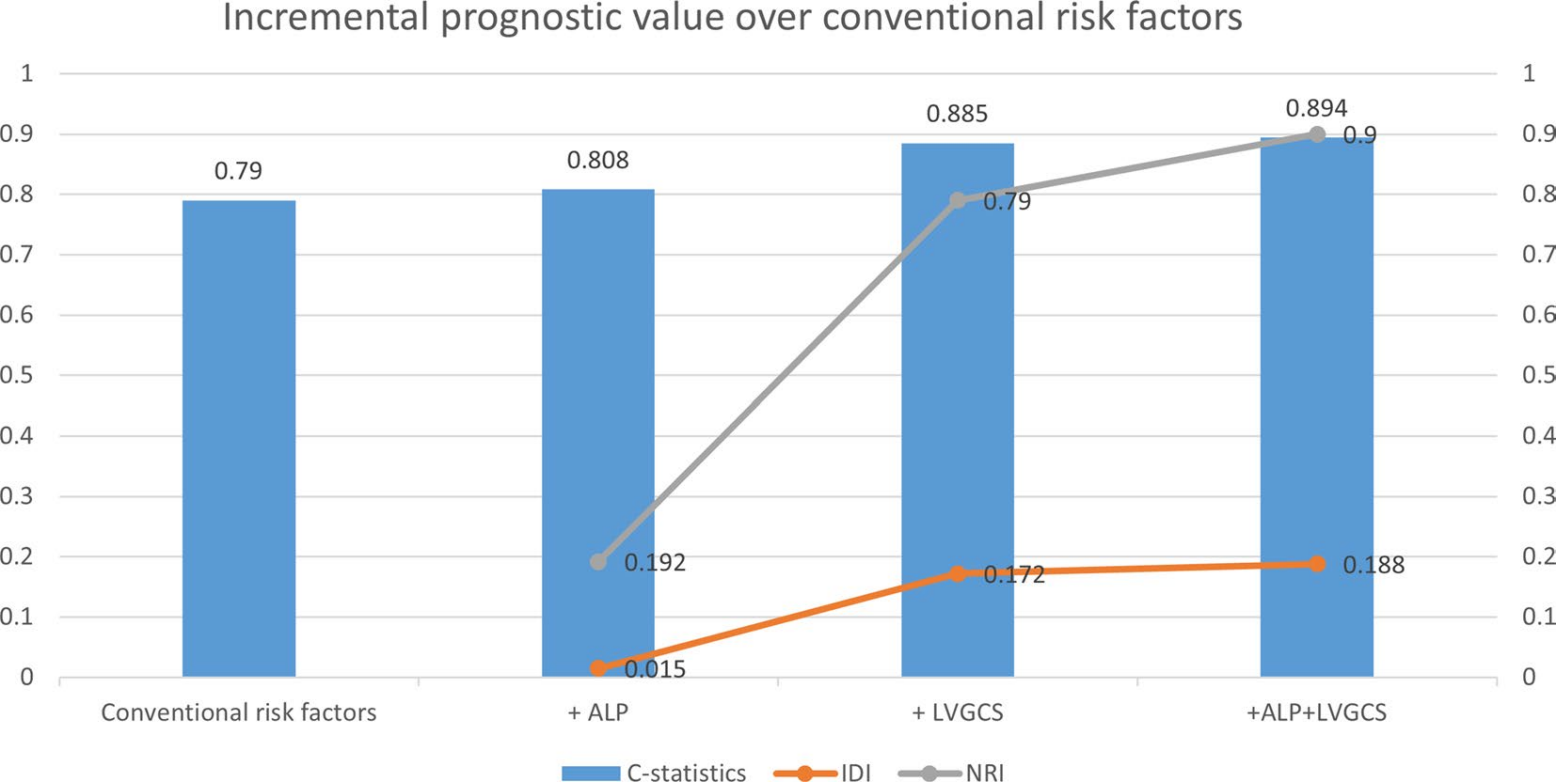
Incremental prognostic value of LVGCS over conventional risk factors. IDI, integrated discrimination improvement; NRI, net reclassification index; ALP, serum alkaline phosphatase; LVGCS, left ventricular global circumferential strain; Conventional risk factors, including age, sex, smoking, hypertension, and diabetes

ROC curve analyses (Fig. 3) indicated that the conventional risk model (including age, sex, smoking, hypertension, and diabetes) demonstrated discriminatory capacity for MACEs (AUC = 0.790, 95% CI 0.688-0.900). The addition of ALP did not substantially alter model discrimination, whereas incorporating LVGCS (alone or with ALP) yielded improved reclassification metrics: IDI = 0.172 and 0.188 (*P* < 0.001), and NRI = 0.790 and 0.900 (*P* < 0.001) (Table 3; Fig. 4).

**Table 3:** Discrimination and reclassification performance for composite clinical outcome

Models	C-statistics		AUC diff		IDI		NRI	
	C-index (95% CI)	*P*	Index (95% CI)	*P*	Index (95% CI)	*P*	Index (95% CI)	*P*
Conventional risk factors	0.790 (0.688-0.900)	< 0.001						
+ ALP	0.808 (0.707–0.915)	< 0.001	0.018 (-0.008-0.043)	0.176	0.015 (-0.006-0.037)	0.164	0.192 (-0.237-0.621)	0.381
+ LVGCS	0.885 (0.807–0.975)	< 0.001	0.095 (-0.026-0.169)	0.008	0.172 (0.086–0.258)	< 0.001	0.790 (0.379–1.201)	< 0.001
+ALP+LVGCS	0.894 (0.810–0.977)	< 0.001	0.104 (-0.029-0.171)	0.006	0.188 (0.099–0.276)	< 0.001	0.900 (0.491–1.309)	< 0.001

AUC, area under the curve; IDI, integrated discrimination improvement; NRI, net reclassification index; CI, confidence interval; ALP, serum alkaline phosphatase; LVGCS, left ventricular global circumferential strain; Conventional risk factors, including age, sex, smoking, hypertension, and diabetes

## Discussions

The principal findings of this study are: (1) During a 19-month follow-up, 37.8% of the CSF patients experienced MACEs; (2) LVGCS and ALP were independently associated with MACEs in CSF patients after adjusting for conventional risk factors; (3) LVGCS may provide incremental prognostic value beyond conventional risk factors. To our knowledge, this is the first study to explore the potential prognostic significance of MS in CSF. These findings may offer a reliable, noninvasive targeted parameter for evaluating prognosis and stratifying cardiovascular risk in CSF patients.

CSF, a manifestation of microvascular dysfunction [14], is associated with significant morbidity, including angina, acute coronary syndrome, and sudden cardiac death [15]. As another endotype of microvascular angina with a different hemodynamic substrate [16], CSF’s pathophysiology remains unclear, involving inflammation, endothelial dysfunction, and microvascular remodeling [3]. Clinically, CSF is characterized by high rates of recurrent chest pain (85–98%) [17, 18]; accordingly, 84.8% of our 336 patients reported chest discomfort, highlighting the substantial clinical burden. This leads to frequent healthcare utilization, with one-third of patients presenting to emergency departments and nearly one-fifth requiring rehospitalization [19, 20]. Though cardiovascular mortality is low (1.2–3.4%) [17, 20], recurrent angina impairs quality of life. Critically, studies indicate that patients with CSF are at high risk for MACEs [18], with case reports documenting severe manifestations such as ventricular arrhythmias and sudden cardiac death [19]. Supporting this, our cohort had an incidence of 37.8% MACEs and 0.9% cardiac mortality. These data collectively emphasize that, despite low mortality, patients with CSF face a high burden of symptoms, recurrent hospitalizations, and increased MACE risk, underscoring the urgent need for objective risk stratification tools to identify high-risk individuals. Our study addresses this unmet need by investigating the prognostic value of MS.

The independent association between ALP and MACEs in our cohort aligns with its established biological roles in vascular calcification, inflammation, and endothelial dysfunction [21–23], which are pathways implicated in CSF pathogenesis. Mechanistically, ALP may promote hydroxyapatite deposition by hydrolyzing pyrophosphate [21] and correlate with inflammatory microcirculatory injury [22, 23], providing a plausible mechanistic basis for the observed association. However, adding ALP to the conventional risk model did not improve predictive performance. This suggests ALP may reflect systemic pathology; its nonspecificity likely limits additional cardiac prognostic value. This highlights the need for more specific, target-organ-focused prognostic indicators for CSF, which our MS observations may provide.

In contrast to ALP, our study showed that STE-derived MS parameter LVGCS was not only independently associated with MACEs but also may exhibit incremental prognostic value beyond conventional risk factors. This potential advantage may stem from MS’s capacity to detect subclinical myocardial dysfunction at the target-organ level. Notably, MS’s prognostic utility varies across cardiac pathologies: in MI, LVGLS reportedly outperforms LVEF and LVGCS in identifying post-reperfusion injury and predicting outcomes, though accuracy may be confounded by acute edema [24]; in hypertrophic cardiomyopathy, MS imaging may may help differentiate hypertrophy etiologies, and LVGLS predicts composite events (strain thresholds remain undefined) [25]; in ventricular arrhythmia ablation patients, LVGLS may predict heart failure mortality but not recurrence [26]; in heart failure, LVGLS predicts aHFpEF/HFrEF outcomes [27], with specificity potentially confounded by comorbidities.

Unlike other cardiac conditions where LVGLS is often the primary prognostic parameter [24, 27], our data indicate LVGCS as the principal MS parameter independently associated with MACEs in CSF after multivariate adjustment. This observation aligns with a prior report noting a particular impairment of LVGCS in CSF [28], suggesting its potential distinct role in this pathology. The pathophysiological basis of this specific association may lie in the nature of microvascular dysfunction. Experimental models indicate that abnormal microcirculatory perfusion preferentially alters circumferential strain [29], likely due to the vulnerability of high-energy-demand, circumferentially oriented mid-myocardial fibers to microcirculatory perfusion deficits. This is consistent with clinical observations linking reduced LVGCS to systemic markers of oxidative stress and endothelial dysfunction [30], which are processes central to microvascular disease and similarly implicated in CSF. Furthermore, LVGCS is recognized for its sensitivity to regional myocardial ischemia [31]. Collectively, these findings support the hypothesis that circumferential fibers may be more sensitive to microvascular disease, making LVGCS a promising specific marker of disease progression. This reinforces its value as a CSF risk stratification tool that complements conventional prognostic factors.

The observed association between LVGCS and MACE risk, together with its potential incremental prognostic value in CSF, positions LVGCS as a candidate for clinical translation. The semi-automated analysis of LVGCS integrates readily into routine echocardiographic workflows without adding extra imaging burden, enabling efficient and reproducible assessment even in community hospital settings. In clinical practice, we recommend incorporating STE-derived LVGCS into routine evaluations for angiographically confirmed CSF patients. This supplementary analysis enhances risk stratification by detecting subclinical microvascular dysfunction that conventional parameters miss, thereby identifying a high-risk subgroup likely to benefit from intensified surveillance and targeted preventive strategies.

This study has several limitations. First, as a single-center retrospective analysis with a modest sample size, the generalizability of our findings to populations with distinct characteristics or healthcare environments remains uncertain. Future prospective multicenter studies are warranted to validate these observations. Second, the retrospective design carries risks of bias, particularly potential recall bias from telephone/outpatient follow-up; this could affect outcome ascertainment reliability. Implementation of standardized protocols (e.g., structured interviews with scheduled in-person assessments) is recommended in subsequent studies. Third, although multivariate adjustments were applied for known confounders, the possibility of residual confounding from unaccounted variables (e.g., socioeconomic factors, lifestyle, or medication adherence) cannot be excluded. Thus, the observed associations should be interpreted as non-causal relationships. Mechanistic investigations and prospective interventional studies would help elucidate potential causal pathways. Finally, future research could explore the integration of LVGCS with supplementary parameters (e.g., coronary microcirculation indices, inflammatory biomarkers, and detailed clinical profiles) to refine predictive models and address residual confounding in diverse clinical contexts.

## Conclusion

This study demonstrates that LVGCS may provide significant incremental prognostic value beyond conventional risk factors for MACEs in CSF. Incorporating the quantification of LVGCS into routine evaluations holds promise as a valuable tool for identifying high-risk CSF patients. Future multicenter studies are warranted to establish validated LVGCS thresholds and determine targeted clinical management approaches for CSF.

## Data Availability

The datasets used and/or analyzed during the current study are available from the corresponding author on reasonable request.
